# The effects of Tai Chi exercise on sleep quality among the elderly: a study based on polysomnographic monitoring

**DOI:** 10.3389/fneur.2024.1304463

**Published:** 2024-03-08

**Authors:** Chao Wang, Tao Jiang, Hansen Li, Guikang Cao, Guodong Zhang

**Affiliations:** ^1^Institute of Sports Science, College of Physical Education, Southwest University, Chongqing, China; ^2^Youth League Committee of Hotan Normal College, HeTian Normal College, Hetian, China; ^3^Physical Education Department, Mianyang High School, Mianyang, China; ^4^Department of Psychology, Southwest University, Chongqing, China; ^5^International College, Krirk University, Bangkok, Thailand

**Keywords:** Tai Chi, sleep quality, polysomnography, sleep disorders, the elderly

## Abstract

**Background:**

Sleep disorders contribute to an increased risk of depression, cardiovascular issues, and various other diseases among older individuals. Consequently, enhancing the sleep quality of this demographic population has become a pressing concern. The objective of this study was to investigate the influence of an 8-week Tai Chi exercise intervention in the sleep quality of older adults.

**Methods:**

Sixty individuals aged 60 years and above, recruited from the community around Southwest University in Beibei District, Chongqing City, were randomly assigned to either a control group (30 participants) or an intervention group (30 participants). The control group adhered to their normal daily routines during the 8-week experimental period, while the intervention group engaged in a 60-min Tai Chi practice three times a week for 8 weeks. Sleep quality was assessed using the Pittsburgh Sleep Quality Index (PSQI), the Insomnia Severity Index (ISI), and the Epworth Sleepiness Scale (ESS). Additionally, the Polysomnographic Sleep Quality Monitoring System (PSG) was employed to monitor the sleep process before and after the Tai Chi intervention.

**Results:**

After the experiment, significant differences were observed in PSQI and IEI scores between the intervention and control groups (*p* < 0.05). In the experimental group, the pre-post comparisons revealed a significant increase in time spent in bed (*p* < 0.05), total sleep time (*p* < 0.05), and non-REM sleep stage 2 (*p* < 0.05).

**Conclusion:**

The findings indicate that Tai Chi exercise may improve subjective reported sleep quality. In addition, Tai Chi exercise may alleviate general drowsiness, extend sleep duration, and optimize the sleep process and structure. Consequently, Tai Chi exercise may be a suitable exercise to improve sleep quality in older individuals.

## 1 Introduction

Sleep is a fundamental physiological need for humans, constituting one-third of an individual's life, that plays a crucial role in maintaining normal growth and development (1), which is closely linked to the overall health (2). In the context of rapid societal development and heightened social competition, the incidence of sleep disorders is escalating. In China, one in three individuals experiences sleep disorders, and globally, sleep disorders affect 9%−15% of the population according to clinical diagnostic criteria. These disorders can lead to various adverse health consequences, including depression, anxiety, mood disorders, hypertension, diabetes, and stroke. They also pose public safety risks such as via road accidents. Sleep disorders have evolved into a significant societal and scientific problem that is affecting the wellbeing of people, thereby requiring immediate attention in China (3).

Across the world, the elderly are the most affected group. Data indicate that 30%−40% of the elderly experience sleep disorders, and 88% of those aged 65 years and above experience various degrees of sleep issues (4). Insomnia and excessive daytime sleepiness are prevalent health issues among the elderly, affecting up to 50% of older adults. A study funded by the National Institute on Aging, involving over 9,000 people aged 65 years and above, revealed that more than half of the participants reported experiencing at least one sleep disorder. Common symptoms found among older adults include difficulty falling asleep, interrupted sleep, early morning awakenings, and excessive daytime sleepiness. These issues contribute to compromised health, reduced physical activity, limitations in daily activities, poor physical functioning, increased depressive symptoms, and increased morbidity and mortality from cardiovascular diseases (5).

Currently, medication, cognitive-behavioral therapy (CBT), and physical exercise are the most common approaches to improving sleep quality. However, medications may have side effects such as gastrointestinal dysfunction, worsening insomnia, obesity (6), and, in extreme cases, even mortality (7). While CBT is effective with minimal adverse effects, it has a drawback of slow efficacy and a prolonged treatment cycle (8). On the other hand, exercise has demonstrated superior effectiveness in regulating sleep. Regular physical exercise is a safe, feasible, positive and effective method that plays a significant role in improving sleep quality in the elderly (9). Regular physical activity contributes to better sleep and reduce daytime sleepiness (10). Aerobic exercise specifically improves sleep quality in older adults, with its impact on sleep through the modulation of psychological and other parameters (11).

Tai Chi, a traditional Chinese martial art, comprises various forms involving slow, coordinated movements, postural alignment, and synchronized deep breathing. Tai Chi is also recognized as a form of Qigong, a traditional Chinese regimen promoting health (12). Characterized by slow body movements and moderate intensity, Tai Chi may be particularly suitable for older adults. Tai Chi has been practiced to improve cardiovascular health and physical fitness (13) as well as to mitigate the decrease in bone mineral density (BMD) caused by aging (14). Furthermore, research indicates that Tai Chi also contributes to alleviate fatigue, anxiety, depressive symptoms, and sleep disorders (13).

Currently, numerous studies collectively imply the effectiveness of Tai Chi in improving sleep health. The observed benefits include improved sleep quality, reduced insomnia (15), and decreased daytime sleepiness (5). Notably, similar benefits appear to exist among older adults (16–19). These findings bolster the idea of Tai Chi as a potential non-pharmacological intervention for improving sleep health among older adults. However, many relevant investigations predominantly relied on subjective measures such as the Pittsburgh Sleep Quality Index (PSQI), the Insomnia Severity Index, and the Epworth Sleepiness Scale. These scales, based on individual perceptions, lack a robust scientific foundation for evaluating detailed sleep conditions.

In this context, polysomnography (PSG) stands out as an objective measurement system that continuously and synchronously monitors over 10 physiological signals, including electrocardiogram (ECG), electroencephalogram (EEG), electromyogram (EMG), electrooculogram (EMG), oculomotorgram (EMG), thoracic respiration, and abdominal respiration throughout the duration of sleep. PSG allows the effective monitoring of the entire duration of sleep and is globally recognized as the gold standard of sleep measurement (20). Currently, PSG primarily serves in the diagnosis of sleep and psychiatric disorders, including tasks such as diagnostic typing of sleep apnea, severity assessment, treatment effectiveness evaluation, diagnostic evaluation of excessive narcolepsy (episodic somnolence, OSAS), and exploration of reactions to the reality of sleep disorders associated with psychiatric conditions (21).

This study aimed to evaluate the influence of Tai Chi exercise on the sleep of elderly individuals, employing both objective and subjective measures. Our research hypothesis is that Tai Chi exercise has the potential to enhance both subjectively perceived and objectively measured sleep conditions.

## 2 Materials and methods

This study constituted a randomized controlled trial aimed at investigating the effects of Tai Chi on sleep conditions in older adults.

### 2.1 Participants

The study participants were recruited from urban communities near the Southwest University School Hospital in Chongqing, China, specifically from Chaoyang Street, Tiansheng Street, Bei Wen Quan and Longfeng Community, spanning from June 2020 to December 2020. This study involved 60 individuals aged between 60 and 79 years who were randomly assigned to either the Tai Chi intervention group (4 men and 26 women) or the control group (4 men and 26 women). General information regarding the elderly individuals in both groups exhibited no significant differences (*P* > 0.05), ensuring baseline comparability.

Table 1 provides a summary of the basic information of the study participants. Upon obtaining informed consent andcompleting baseline assessments, participants were randomly assigned to the intervention groups in a 1:1 ratio using computer-generated random numbers. The randomization process involved no stratification or blocking factors. Participants remained unaware of their group assignments until the baseline assessment was concluded. Importantly, the individuals responsible for generating the randomization plan refrained from participating in the screening or testing processes. Five participants in the control group withdrew voluntarily for personal reasons.

**Table 1:** Basic information of the study participants.

**Groups**	**Gender**	**Age**	**Educational attainment**	**Number of people**
Intervention group	Man 13.3%; Woman 87.7%	66.0 ± 4.97	10.700 ± 2.1838	30
Control participants	Man 13.3%; Woman 87.7%	69.1 ± 4.56	9.58 ± 2.9268	30

### 2.2 Inclusion criteria

The inclusion criteria for the study participants were as follows: (1) older adults aged 60 years and above; (2) scores equal to or greater than 26 on the Brief Mental State Examination (MMSE) scale; (3) the absence of illnesses impacting postural control, such as vestibular sensory system disorders or visual system disorders; (4) no clinical neurological and psychiatric disorders, with participants not currently using antidepressant, anxiety, or related medications; and (5) understanding and voluntarily agreeing to participate in the experiment while meeting the eligible criteria. After screening on-site and through WeChat, a total of 60 older adults were included in the experimental study.

### 2.3 Exclusion criteria

This study listed the following exclusion criteria: (1) individuals involved in any other form of sport during the intervention period; (2) participants who had already undergone other types of sleep improvement interventions; (3) individuals unable to arrive at the designated location for the intervention within a fixed timeframe; (4) individuals using sleep medications; and (5) elderly individuals with a long-term Tai Chi practice history.

### 2.4 Dropout criteria

Participants were considered for dropout if they met the following criteria: (1) participants with two or more consecutive absences during the intervention period; (2) individuals with <20 total interventions; (3) participants engaging in other forms of physical activity during the intervention period; (4) those experiencing adverse physical reactions during the intervention process; and (5) participants voluntarily withdrawing from the intervention trial, completing their involvement in the trial.

### 2.5 Procedure

**Figure 1:**
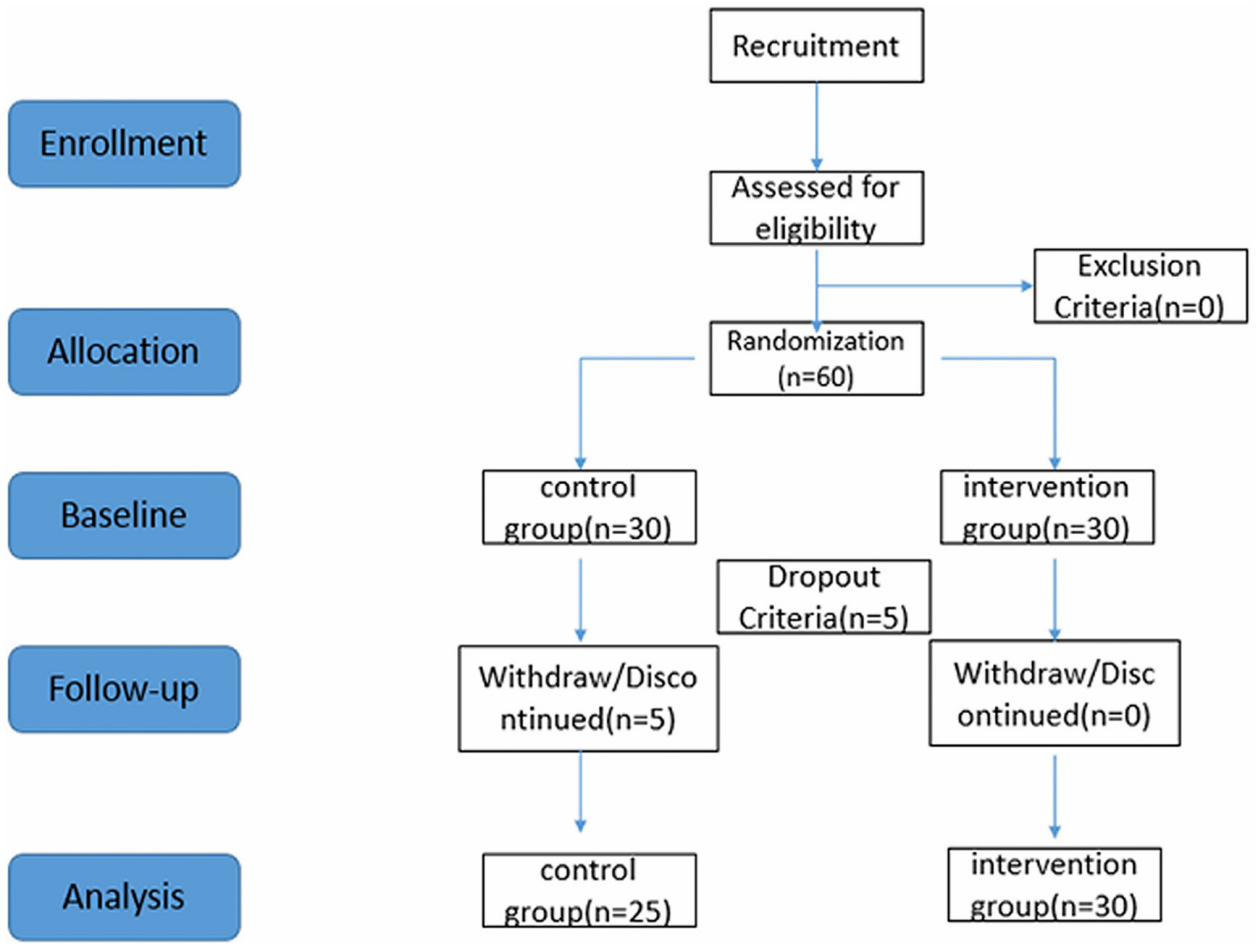
The experimental procedure and timeline.

In this study, the elderly participants were randomly assigned to either the control group or the intervention group. Both groups underwent assessments of their subjective sleep, with specific post-intervention measures conducted accordingly. The subjective sleep quality of both the intervention and control groups was evaluated 1 week before the experiment and 1 week after its completion. Throughout the 8-week experimental period, the control group maintained regular physiological activities and living habits. Meanwhile, in the intervention group, Polysomnographic Sleep Quality Monitoring System was employed to monitor the all-night sleep of each older adult 1 week before the experiment and again 1 week after the intervention. The experimental procedure is shown in Figure 1.

### 2.6 Schedule

The Tai Chi intervention group practiced a 24-pattern Tai Chi exercise three times a week (Monday, Wednesday, and Friday) for 60 min during the 8-week experimental period from 7:00 a.m. to 8:00 a.m. In the second phase, due to the late dawn time during the winter and the safety concerns, the practice time was rescheduled to 7:30 a.m. to 8:30 p.m. A check-in will be carried out prior to each Tai Chi exercise practice to ensure the attendance of the experimental participants and improve the quality of the intervention.

### 2.7 Experimental setting

We offered flexibility in scheduling for participants to engage in Tai Chi exercise under our guidance within specified timeframes. Participants had the liberty to choose a convenient time slot to participate in the 24-pattern Tai Chi exercise based on their personal schedules. Meanwhile, the control group adhered to their original lifestyle habits, refraining from any form of physical exercise, and engaged in online health lectures conducted once every 2 weeks.

For the experimental intervention group, we employed two experienced martial arts teachers (one man and one woman) with more than 5 years of teaching experience. The teaching approach involved one teacher and one assistant to ensure that participants could grasp the fundamental principles of Tai Chi. The teachers addressed related questions from the elderly participants after each session. Considering the lower physical fitness and organ function of elderly, the difficulty of certain complex and potentially hazardous movements (e.g., right stomping, left and right downward movement) was adjusted based on their actual condition, prioritizing safety while facilitating skill mastery and practice.

Over the course of 8 weeks, the intervention group mastered the 24 simplified Tai Chi styles. Wushu teachers thoughtfully organized the teaching content and practice duration to align with the participants' abilities and acceptance levels. This approach aimed to help the elderly master all movements early on, gradually progressing to practicing the complete sets of movements. The detailed course content is outlined in Table 2.

**Table 2:** Tai Chi program content.

	**Preparatory activity (15 min)**	**Main exercise (45 min)**	**Tidying up activity (10 min)**
Course content	1. Unarmed gymnastic exercises; 2. Arrow-quiver, stroke, squeeze, press, and other individual movement exercises	1. Learn 24 Simplified Tai Chi Forms, including: I. Starting; II. Wild Horse Separating Mane; III. White Crane Shining Wings; IV. Knee-wrapping and Reversing Steps; V. Hand-waving Pipa; VI. Inverted Rolling Humerus; VII. Left Range Sparrow's Tail; VIII. Right Range Sparrow's Tail; IX. Single Whip; X. Clouded Hands; XI. Single Whip; XII, Right down independent; XVIII, left and right shuttle; XIX, the bottom of the sea needle; XX, flash through the arm; XXI, turn around to move blocking whacking; XXII, such as sealing like a closed; XXIII, the cross hand; XXIV, the closing trend of 24 technical action. 2. After completing the corresponding learning tasks, carry out before and after action coherence practice.	1. Static stretching of the back of the thighs at a bent over position; 2. Anterior thigh stretch; 3. Standing shoulder shake
Role of exercises	1. Unarmed gymnastics practice process so that the practitioner in the exercise before the whole body muscle to get active mobilization effect. 2. The practice of individual technical movements can strengthen the mastery and understanding of technical movements of the elderly in the process of practice.	1. Practicing 20-Style Simplified Tai Chi can maintain a state of calmness and peace of mind and consciously regulate the body to achieve the most suitable state of relaxation. The slow continuation of the exercises is good for the blood circulation of the elderly and promotes the mobility of the joints, and the coherent movement of the whole set of exercises can enhance the coordination of the body. 2. The related pace of movement can enhance the lower limb strength of the elderly to a certain extent.	1. Single leg support, opposite side of the heel on the ground, hook the back of the foot hands to the front of the foot (the head of the upper limb can not be bent downward and look forward). Relax the calf muscles and the back of the thigh muscles. 2. Hold the pole with one hand and pull the same side of the foot toward the hip with the same side hand. Relax the rectus femoris and anterior thigh muscles. 3. Stand with your legs parallel to each other and shake your shoulders up and down naturally. Relax the shoulder and arm muscles on both sides.

### 2.8 Measurements

#### 2.8.1 Questionnaire

In this study, the Pittsburgh Sleep Quality Index (PSQI), the Epworth Sleepiness Scale (ESS), and the Insomnia Severity Index (ISI), developed by Buysse et al. (22), psychiatrists at the University of Pittsburgh, USA, were utilized to assess the sleep quality in the elderly population.

#### 2.8.2 Polysomnographic sleep monitoring system (PSG)

For this study, a polysomnographic monitoring system of the model E-Series EEG/PSG Recording System, manufactured by Compumedics Ltd., Australia, was employed.

1. Test conditions: All-night sleep monitoring was conducted under conditions similar to those experienced by the patients, ensuring a setting free of interference, quiet, and conducive to sleep.
2. Intervention method: Polysomnographic sleep monitoring was carried out on all patients in the intervention group 1 week before and 1 week after the experiment. To ensure the smoothness of the monitoring process, we visited the patient's home 1 h before the experiment to communicate with the them. Through verbal communication, we emphasized the safety and timeliness of the monitoring process, ensuring that it remains uninterrupted by external factors. The monitoring duration typically ranged from 22:00 h on the same day to 07:00 hon the next day.
3. Monitoring indicators: Polysomnography primarily observes the sleep process, sleep maintenance, sleep structure, and related sleep indicators.

Specific indicators for sleep include the following factors:

Time in bed refers to the total duration recorded from the beginning to the end of monitoring.
Sleep time refers to the duration recorded from the beginning of the patient's sleep to their awakening.
Total sleep time is calculated as the patient's sleep time minus their awakening time.
Awakening time refers to the total time of the patients being awake during the course of sleep monitoring.
N1 time refers to the total time of entry into the first stage of non-rapid-eye-movement sleep, representing light sleep.
N2 time refers to the total time from the end of the first stage of non-REM sleep to the second stage.
N3 time refers to the total time from the end of the second stage of non-REM sleep to entering deep sleep.
REM stage refers to the time spent in REM sleep.
Sleep latency refers to the sum of the time of SLL sleep latency, N2 latency time, and N3 latency time.
Sleep efficiency is calculated as the ratio of the time of sleep to the time of being in bed.
Sleep duration refers to the number of awakenings, ratio of total awakening time to total sleep time.
Sleep Structure: (a) SLL Latency Time; (b) Sleep Latency Time; (c) N1 Latency Time; (d) N2 Latency Time; (e) REM Latency Time; (f) Non-rapid Eye Sleep (N1%, N2%, N3%, and REM%) as a percentage of total sleep time.

### 2.9 Statistical analysis

This study was structured as a randomized controlled trial, focusing on comparing outcomes between the experimental and control groups after the intervention. Consequently, the independent samples *t*-test was utilized to compare data collected in the two groups after the intervention. However, due to COVID-19 restrictions preventing the collection of objective data (using PSG) in the control group, a paired-samples *t*-test was employed to assess whether the objective data significantly changed from baseline to the post-intervention test in the intervention group. A two-tailed *p*-value < 0.05 was deemed statistically significant.

## 3 Results and analyses

### 3.1 A comparison of PSQI, ISI, and ESS scores between groups

The PSQI and ISI scores of the intervention group were significantly lower than those of the control groups after the intervention. However, no significant difference between the two groups was found in terms of ESS scores.

### 3.2 A comparison of objective sleep assessments in the intervention group

#### 3.2.1 A comparison of sleep processes in the intervention groups

In the intervention group, the time in bed (TIB) (*p* < 0.05), time in sleep (TST) (*p* < 0.05), and non-REM sleep stage 2 (N2) (*p* < 0.01) were significantly improved after the intervention. The time of non-REM sleep stage 3 (N3) was reduced. There were some increases in the data of the other groups, but none of them were statistically significant (*p* > 0.05) (Table 3).

**Table 3:** Comparison of PSQI, ISI, and ESS scores on subjective scales after the experiment.

**Type of scale**	**Periods**	**Control groups**	**Intervention group**	***t***	***p***
PSQI	Pre-intervention	5.885 ± 3.7982	6.077 ± 4.010	−0.511	0.611
Post-intervention		6.039 ± 2.9730	4.885 ± 4.430	2.054	0.044^*^
ISI	Pre-intervention	3.923 ± 6.1769	4.466 ± 4.1591	−0.344	0.731
	Post-intervention	4.555 ± 0.8932	3.269 ± 5.0244	2.652	0.010^*^
ESS	Pre-intervention	4.115 ± 4.5460	4.462 ± 4.1591	−0.769	0.444
	Post-intervention	4.192 ± 4.1859	4.385 ± 2.4013	−0.092	0.926

^*^Indicates a *p-*value of < 0.05 for the Control and Intervention Group comparisons.

**Figure 2:**
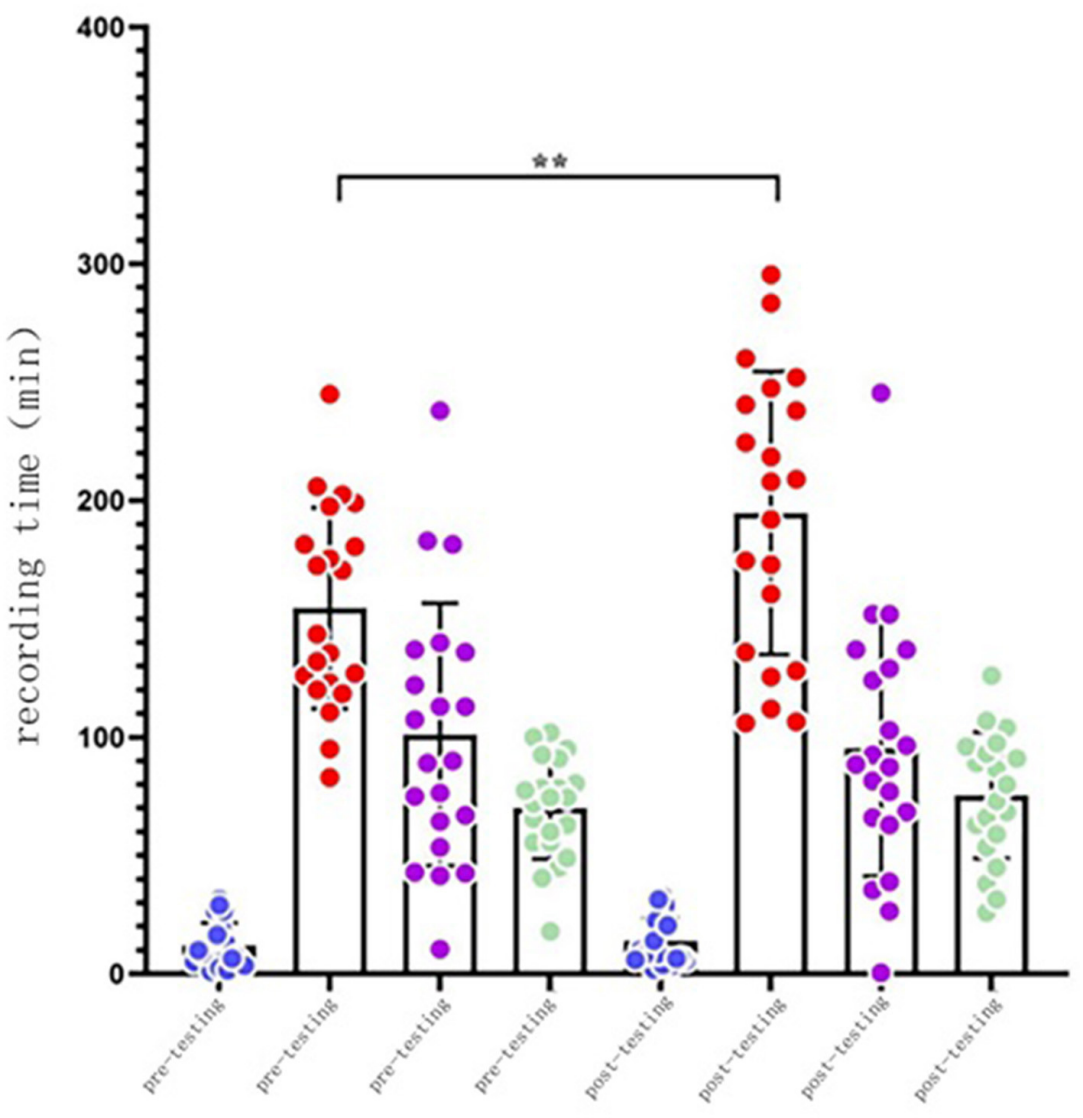
Comparison of sleep progress before and after intervention in the intervention group.

As illustrated in Figure 2, the N1 sleep time basically remained unchanged. After the intervention, N2 sleep time substantially increased, demonstrated with a scatterplot of the N2 sleep time in the elderly, but the sleep time was significantly increased. N3 sleep time experienced a small decline in the elderly, and deep sleep time was more aggregated, while REM sleep time increased.

#### 3.2.2 Comparison of sleep persistence in the intervention group

The waking time of the experimental group significantly increased after the intervention (*p* < 0.05), and there was no significant difference in other indicators throughout the intervention (*p* > 0.05) (Table 4).

**Table 4:** Comparison of sleep processes before and after the experiment in the intervention group.

**Experimental group**	**Bed time**	**Total sleep time**	**N1 time**	**N2 time**	**N3 time**	**Sleep latency**	**REM latency**
Pre-intervention/min	526.1 ± 36.6	337.7 ± 54.1	11.9 ± 9.6	154.5 ± 42.4	101.1 ± 55.5	50.4 ± 47.5	145.6 ± 63.7
Post-intervention/min	567.3 ± 51.9	379.5 ± 62.2	13.8 ± 9.8	194.8 ± 59.8	95.3 ± 54.0	48.6 ± 45.6	155.1 ± 66.5
t	−2.814	−2.760	−1.497	−3.365	0.758	0.115	−0.507
p	0.011^*^	0.012^*^	0.149	0.003^*^	0.457	0.909	0.618

*P* < 0.05^*^ significant difference; *p* < 0.01^**^ highly significant difference.

#### 3.2.3 Comparison of sleep structure in intervention groups

The non-rapid eye movement (NREM) sleep time (*p* < 0.01) and the percentage of non-rapid eye movement (NREM) sleep stage 2 of the intervention group were significantly increased (*p* < 0.05) after the intervention. However, no other significant change was observed (Table 5).

**Table 5:** Comparison of sleep persistence before and after the experiment in the intervention group.

	**Pre-intervention**	**Post-intervention**	***t***	***p***
Awake time	188.5 ± 66.9	187.9 ± 59.4	0.038	0.970
Number of times awake	18.9 ± 8.0	22.8 ± 9.4	−2.679	0.014^*^
Percentage of awakening	0.35 ± 0.11	0.33 ± 0.09	0.929	0.364
Number of sleep entries into REM	5.6 ± 3.3	5.3 ± 3.2	0.542	0.594

*P* < 0.05^*^ significant difference; *p* < 0.01^**^ highly significant difference.

## 4 Discussion

This study aimed to evaluate the influence of Tai Chi exercise on sleep quality of elderly population using both subjective and objective measures. Our research hypothesis was broadly verified, indicating that Tai Chi exercise may positively change both subjective and objective sleep quality among the normal elderly population. Specifically, scores on the Pittsburgh Sleep Quality Index and Insomnia Severity Index, but not on the Narcolepsy Scale, were significantly decreased following the exercise intervention (based on the comparisons between groups). Although the polysomnography-monitored data were only restricted to the intervention group, significant improvements were observed in time in bed, total sleep time, N2 time in bed, N2 time, and awakening time, indicating significant changes in the sleep structure.

### 4.1 Effect of Tai Chi on sleep quality

Our findings based on questionnaire are in line with those of previous studies. For example, we found lower ISI scores in the intervention group, which supports a systematic review, suggesting that Tai Chi exercise can improve sleep quality in healthy elderly individuals and serve as an adjunct for those with insomnia (23). Meanwhile, we also found lower PSQI scores in the Tai Chi intervention group post-intervention, which supports the previous review that Tai Chi improved self-rated sleep quality among older people (24). Taken together, these findings have highlighted the value of Tai Chi exercise for health promotion of older people.

The effects of Tai Chi exercise on sleep may be explained using the following perspective. On the one hand, Tai Chi may increase the volume of daily physical activity, leading to additional energy consumption and ultimately promoting sleep quality (25). On the other hand, Tai Chi may reduce negative emotional symptoms, thereby promoting sleep health. For example, many studies have suggested that Tai Chi may reduce depression (26, 27), which is possibly accompanied by improved sleep quality (28). Furthermore, Tai Chi exercise also involves the regulation of vegetative nervous system, leading to a decrease in the excitability of sympathetic nerves and an organized cerebral cortex. As a consequence of these adjustments, the quality of sleep can be possibly improved (29).

### 4.2 Effects of Tai Chi on sleep processes

The sleep process in individuals over 60 years of age consists of the N1, N2, and N3 sleep stages, as they do not experience the fourth stage of deep sleep. We found that Tai Chi exercise increased total sleep time, prolonged duration of N2 sleep stage, and reduced sleep latency. These findings align with the results of the studies by Irwin et al. (30) and Fan et al. (31), indicating that traditional Chinese medicine (TCM), Tai Chi, and western medicines can improve sleep structure, sleep process, and sleep maintenance in patients with depressive sleep disorders of the heart-spleen deficiency type. Notably, the study conducted by Fan et al. focused on patients with depression and sleep disorders, whereas the current study investigated normal elderly individuals.

It is widely acknowledged that exercise can influence sleep (32), and assessing the effects of physical activity on sleep using polysomnography provides an objective perspective, helping to eliminate the influence of subjective factors. For instance, low-intensity physical activity has been associated with objective benefits related to deep sleep parameters in older adults (33), and interventions involving different physical activity methods, such as treadmill exercise, have shown positive effects on insomnia (34). Our study, from the perspective of Tai Chi exercise, again confirms the effectiveness of physical exercise in improving objectively measured sleep conditions. Nevertheless, it should be noted that our objective measures were carried out only in the Tai Chi intervention group due to the COVID-19 pandemic; hence, the findings were derived from pre-post comparisons. These comparisons could be affected by time-related external factors, such as seasonal changes in human physiology; therefore, our findings must be taken into consideration with caution.

### 4.3 Research limitations

Our sample was recruited based on the maximum effort given the available resources; however, the recruitment of 60 participants is still insufficient. Based on our analysis, the achieved statistical power was 0.48 (independent samples *t-*test) and 0.75 (paired samples *t-*test) (based on a two-tailed test with an effect size of 0.5). This flaw may have reduced our ability to detect effects, increased the type I error rate, and limited the generalizability of our findings. Therefore, future experiments with larger samples are still needed to replicate our findings. In the results of polysomnography, testing was conducted only before and after the intervention in the intervention group. Additionally, the shorter intervention period and lower frequency of intervention may have contributed to the data sensitivity issues. Further in-depth studies are required to explore the effect of Tai Chi exercise on the sleep process of the normal elderly population.

## 5 Conclusion

We conducted a controlled intervention to examine the influence of Tai Chi on the sleep conditions of older adults. Although we did not recruit a satisfactory sample size due to the COVID-19 pandemic and failed to conduct all planned tests, our preliminary results provide initial evidence of the positive effects of Tai Chi on improving sleep quality in older adults. The improvement in objective sleep measures based on instrument measurements complement previous questionnaire surveys regarding this topic.

## Data availability statement

The raw data supporting the conclusions of this article will be made available by the authors, without undue reservation.

## Ethics statement

The studies involving humans were approved by the Ethics Review Board of University. The studies were conducted in accordance with the local legislation and institutional requirements. The participants provided their written informed consent to participate in this study.

## Author contributions

CW: Writing – original draft. TJ: Writing – review & editing. HL: Writing – review & editing. GC: Writing – review & editing. GZ: Writing – review & editing, Supervision.
